# Study on Association of Pentraxin 3 and Diabetic Nephropathy in a Rat Model

**DOI:** 10.1155/2018/8968573

**Published:** 2018-03-13

**Authors:** Xuehai Chen, Jiao Luo, Minmin Wu, Zhuo Pan, Yue Xie, Hongwei Wang, Bicheng Chen, Hong Zhu

**Affiliations:** ^1^Department of Surgery, The First Affiliated Hospital of Wenzhou Medical University, Wenzhou, Zhejiang 325000, China; ^2^Zhejiang Provincial Top Key Discipline in Surgery, Wenzhou Key Laboratory of Surgery, Department of Surgery, The First Affiliated Hospital of Wenzhou Medical University, Wenzhou, Zhejiang 325000, China; ^3^Department of Endocrinology, The First Affiliated Hospital of Wenzhou Medical University, Wenzhou, Zhejiang 325000, China

## Abstract

Diabetic nephropathy (DN) is a serious microvascular complication of diabetes. Compared with other therapies for diabetic patients, islet transplantation can effectively prevent and reverse diabetes-induced microvascular disease, such as diabetic retinopathy and nephropathy. PTX3 is the only long pentraxin that can be detected in renal tissue. In this study, we investigated the expression of PTX3 when early DN was reversed after islet transplantation. *Methods*. Diabetes was induced in rats by injecting streptozotocin (STZ). Twelve weeks later, the diabetic rats were divided into 2 groups: the islet transplantation group (IT) and the diabetic nephropathy group (DN). Renal injury, renal function, and the expression of PTX3 in the plasma and the kidneys were assessed with urinalysis, immunohistochemical staining, and Western blot, respectively. *Results*. The expression of PTX3 in the kidney was significantly decreased in the DN group but increased in the IT group because of the reversal of DN. *Conclusions*. Our data showed that the level of PTX3 in renal tissue is closely related to renal injury in DN. This may be used to quantify the extent of renal injury in DN, provide a potential early indicator of renal tubular injury in early DN patients, and assess DN clinical progression.

## 1. Introduction

In recent decades, the prevalence of diabetes in Chinese adults has risen to 9.7% [1]. Diabetic nephropathy (DN), a serious microvascular complication of diabetes, remains the main cause of end-stage renal disease. The major characteristic changes of DN include glomerular mesangial expansion, glomerular basement membrane thickening, progressive renal dysfunction, and persistent proteinuria [2]. Current management of hyperglycemia around the world depends on the lifetime injection of insulin. However, lifetime insulin administration has a high cost to patients, an elevated risk of hypoglycemia caused by improper insulin use, and can cause skin hypersensitivity reactions [3]. Previous studies showed that strict blood glucose control, the administration of angiotensin II receptor blockers (ARB), lipid lowering, and so forth can delay but not reverse the progress of DN. In 1990, Tzakis et al. reported the successful transplantation of isolated islet cells into diabetic patients for the first time [4]. Islet transplantation, a new treatment in modern society, permits more effective blood sugar control and better patient economic performance. More importantly, islet transplantation can effectively prevent and reverse diabetes-induced microvascular diseases, such as diabetic retinopathy and nephropathy [5, 6]. Our previous work found that in a rat model of early diabetic nephropathy, islet transplantation could stop and reverse the progression of diabetic nephropathy [7].

Pentraxin 3 (PTX3), a member of the long pentraxin group, has a structural homology with short pentraxins such as C-reactive protein (CRP) and serum amyloid P component (SAP) [8]. However, in contrast to CRP and SAP, which are produced in the liver, PTX3 is synthesized by extrahepatic tissues and cells in response to inflammatory stimulation, such as by interleukin 6 (IL-6) [8, 9]. PTX3 is composed of eight identical protomer subunits by disulfide bonds and is a member of the highly conserved pentraxin superfamily. PTX3 is produced and released by multiple different cell types, such as endothelial cells, monocytes/macrophages, skeletal muscle, and vascular smooth muscle cells, in response to tissue injury or inflammation [10]. In prior studies, it was demonstrated that as a modulator of inflammatory processes, PTX3 was involved in angiogenesis, atherosclerotic lesions, and extracellular matrix formation [11]. It has also been reported that PTX3 can control the interaction between dendritic cell (DC) maturation and apoptotic cells and then participate in the scavenging of apoptotic cells in the body [12].

PTX3 is the only long pentraxin that can be detected in renal tissue [13]. PTX3 mRNA is expressed in normal proximal renal tubular epithelial cells [10] but is marginally present in normal human glomeruli [13]. The expression of PTX3 was increased in renal endothelial cells in an ischemia-reperfusion injury model [14] and in the glomerular mesangial and endothelial cells of patients with type I membranous glomerulonephritis or IgA nephropathy [13]. However, in renal biopsies of patients with diffuse proliferative lupus nephropathy, membranous glomerulonephritis, or focal segmental glomerulosclerosis, the expression of PTX3 in the glomeruli was almost negative [13]. Instead, in the above renal diseases, PTX3 was expressed in the renal interstitial and tubules. Only one study on the association between PTX3 and DN is currently available, which reported that the expression of PTX3 was remarkably decreased in experimental diabetic nephropathy compared with controls [15].

In this study, we evaluated the expression of PTX3 and other related renal injury parameters in the renal tissue of diabetic rats with early diabetic nephropathy. Furthermore, we investigated the expression of PTX3 when early DN was reversed after islet transplantation.

## 2. Materials and Methods

### 2.1. Animal Model and Groups

A total of 35 male Wistar rats weighting 250–300 g were purchased from the Experimental Animal Center of Wenzhou Medical University. All rats were housed with a 12 h light/dark cycle at 24°C and provided with food ad libitum for a week before the beginning of experimental procedures. All animal experiments were approved by the Wenzhou Medical University Management Committee for Medical Laboratory Animal Sciences. Twelve rats were used as islet donors, 6 rats were used as normal controls (NC group), and the others (*n* = 17) received a single intraperitoneal injection of streptozotocin (STZ) (55 mg/kg of body weight, Sigma-Aldrich, St. Louis, MO, USA) in sodium citrate buffer (pH 4.5) after fasting for 12 hours. One week after injection, blood was taken from the tail vein for 3 consecutive days to measure plasma glucose concentration with an AccuChek glucometer (Roche Diagnostics, Indianapolis, IN). When all blood plasma glucose concentrations were ≥16.67 mmol/L, the diabetic rat model was established. After 12 weeks, the early diabetic nephropathy rat model was defined on the basis of urine ACR and protein-to-creatinine ratio. The diabetic nephropathy rats were randomly divided into two groups and studied 4 weeks later: rats in the untreated group (DN group, *n* = 6) which did not receive treatment and in the islet-transplanted group (IT group, *n* = 6) where rats received an islet transplantation.

### 2.2. Islet Isolation and Purification

This method was described in a previous study [16]. Donor rats were anesthetized with 5% isoflurane mixed with 0.5 L/min oxygen, followed by a reduced concentration of isoflurane (2%) to maintain anesthesia. After disinfection of the abdominal skin, a U-incision was cut in the abdomen, and the pancreas was completely exposed. The entrance of the common bile duct into the duodenum was located and ligated, and 10 mL of collagenase V (2 mg/mL, dissolved in Hank's solution) was injected into the common bile duct using retrograde intubation. After complete perfusion, the expanded pancreas was carefully transferred to a sterile 50 mL centrifuge tube, to which 2 mL of precold V collagenase solution was added in advance, and then was digested for 3 min at 37 ± 0.5°C. The pancreatic tissue was transferred into a 50 mL centrifuge tube, and ingestion was terminated by adding 40 mL of Hank's solution. After gradient centrifugation, the islet cells were manually pick up under a microscope and cultured in RPMI-1640 (Gibco, Carlsbad, CA, USA) containing 10% fetal bovine serum (FBS; Gibco, Invitrogen Inc., USA) at 37°C and 5% CO_2_ for 6 hours.

### 2.3. Preparation of Islets before Transplantation

The islet cells were randomly divided into two parts. One part was stained with fluorescein diacetate-propidium iodide (FDA-PI) to detect islet cell activity. After the other part was counted, these islets were aspirated into a 1 mL syringe connected to P50 polyethylene tubing and then transferred to the head end. Total islet equivalents (IEQ) were calculated according to the appropriate formula [17]. Approximately 1000–1200 IEQ of purified islets were obtained.

### 2.4. Islet Transplantation under the Kidney Capsule

This method was previously reported by Napoli et al. [18]. Recipient rats were anesthetized with isoflurane, and their left flank was shaved and disinfected with 75% alcohol and iodophor. The kidney was gently squeezed out through a small lumbar incision, and polyethylene tubing was inserted under the kidney capsule. The islets in the tubing were pushed gently, and the tubing was removed until the islets were completely transplanted under the kidney capsule.

### 2.5. Urine, Blood, and Tissue Sampling

At weeks 12 and 16, the 24 h urine sample was collected with a rat metabolic cage. Urine protein concentration was measured using the sulfosalicylic acid precipitation method, and the creatinine concentration was assessed with a creatinine assay kit (Bioassay Systems, Hayward, CA). Albuminuria was measured using a rat albumin-specific ELISA kit (Exocell Laboratories, Philadelphia, PA). Commercial assay kits for serum creatinine (Scr) and blood urea nitrogen (BUN) were purchased from the Nanjing Jiancheng Institute of Biotechnology (Nanjing, China). Scr and BUN concentrations were measured according to the protocols provided by the manufacturers. The nonfasting blood glucose and body weight of members of each group were measured twice per week. At week 16, all rats were sacrificed after anesthesia with isoflurane. Select kidney tissue was fixed in 4% paraformaldehyde for histopathologic staining. The remaining tissue was stored in liquid nitrogen. After gradient alcohol dehydration and xylene transparence, the kidney tissue was incorporated into a wax block and cut into 4 *μ*m sections.

### 2.6. Histopathology

Kidney tissues fixed in formaldehyde were dehydrated, embedded in paraffin, and cut into 4 *μ*m thick sections. Sections treated with hematoxylin-eosin (HE) staining were observed with a light microscope at a high magnification.

### 2.7. Immunohistochemical Staining

For immunohistochemical analysis, paraffin-embedded kidney tissue sections (4 *μ*m thick) were used. Sections were placed on glass slides, deparaffinized, and hydrated with xylene and graded alcohol. Sections were preincubated in a boiling sodium citrate buffer for antigen retrieval. Primary antibodies (1 : 200) for PTX3 (Bioworld Technology Inc., St. Louis Park, MN, USA), *α*-SMA (Abcam, Cambridge, MA, USA), caspase 3 (Abcam, Cambridge, MA, USA), and TGF-*β*1 (Abcam, Cambridge, MA, USA) were added and incubated at 4°C overnight. The tissue sections were then incubated for 1 h with HRP-conjugated anti-rabbit IgG. After DAB and hematoxylin staining, tissue sections were viewed under an optical microscope. The average optical density was calculated by Image-Pro Plus 6.0 image analysis software (Media Cybernetics, Silver Spring, MD).

### 2.8. Western Blot Analysis

Western blot analysis was performed to measure the protein expression of PTX3, Bax, Bcl-2, NPHS-2, and GAPDH. Kidney tissues were lysed with RIPA buffer, and protein concentrations were measured with a BCA assay kit (Beyotime Biotechnology, Jiangsu, China) according to the manufacturer's protocol. Proteins were separated with 12% sodium dodecyl sulfate-polyacrylamide gels (SDS-PAGE), then transferred to a polyvinylidene fluoride (PVDF) membrane (Millipore Corporation, Bedford, MA, USA). The membrane was blocked with 5% fat-free milk for 1 h at room temperature and then further incubated overnight at 4°C with the following primary antibodies: PTX3 (Bioworld Technology Inc., St. Louis Park, MN, USA, 1 : 1000), Bax (Cell Signaling Technology Inc., Danvers, MA, USA, 1 : 1000), Bcl-2 (Cell Signaling Technology Inc., Danvers, MA, USA 1 : 1000), NPHS-2 (Abcam, Cambridge, MA, USA), and GAPDH (Bioworld Technology Inc., St. Louis Park, MN, USA, 1 : 10,000). The membranes were then incubated with horseradish peroxidase- (HRP-) conjugated secondary antibody (goat anti-rabbit IgG; Bioworld Technology Inc., St. Louis Park, MN, USA) diluted to 1 : 10,000 at 37°C for 1 h. Protein bands were detected using Image Lab Software (Bio-Rad Laboratories Inc., Berkeley, CA, USA).

### 2.9. Enzyme-Linked Immunosorbent Assay (ELISA)

The levels of plasma ptx3 in each groups were measured according to the manufacturer's protocol (USCN, Wuhan USCN Business Co. Ltd.). The absorbance was measured at 450 nm using a Varioskan Flash™ multimode microplate reader (Thermo Fisher Scientific, Waltham, MA, USA).

### 2.10. Statistical Analysis

All data were analyzed using GraphPad Prism 5 (GraphPad software, version 5, San Diego, CA, USA), and values are expressed as the mean ± standard deviation. Multiple comparisons between groups were performed with a one-way ANOVA. *p* < 0.05 was considered significant.

## 3. Results

### 3.1. Evaluation of an Early Diabetic Nephropathy Model in Rats

**Figure 1:**
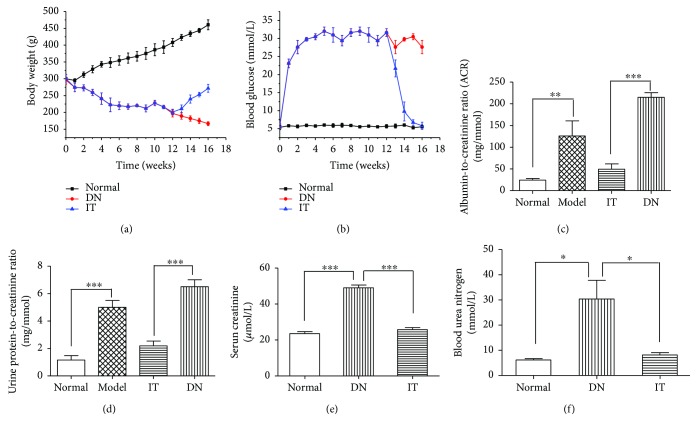
Changes in rat blood glucose, body weight, and urine protein-to-creatinine ratio and urine microalbumin-to-creatinine ratio. (a) Changes in body weight. (b) Rat blood glucose for each group. (c) Urine microalbumin-to-creatinine ratio. (d) Urine protein-to-creatinine ratio. (e) Serum creatinine. (f) Blood urea nitrogen. ^∗^*p* < 0.05, ^∗∗^*p* < 0.01, and ^∗∗∗^*p* < 0.001.

As shown in Figures 1(a) and 1(b), the blood glucose of the DN group injected with STZ increased rapidly and maintained levels of 25 mmol/L after 1 week. Compared with the normal group, the blood glucose of the DN group changed significantly, and body weight continued to decrease with time. Figures 1(c) and 1(d) show that the urine protein-to-creatinine ratio (6.512 ± 0.512 mg/mmol) and the urine microalbumin-to-creatinine ratio (215 ± 13.2 mg/mmol) in the DN group increased significantly compared with those of the normal group (1.159 ± 0.32 mg/mmol and 24.275 ± 3.8 mg/mmol, resp.) (*p* < 0.01; *p* < 0.001). Changes in rat blood glucose, body weight, and urine protein-to-creatinine and urine microalbumin-to-creatinine ratios demonstrate that a model of early DN was successfully established [19, 20].

### 3.2. Evaluation of Islet Transplantation

**Figure 2:**
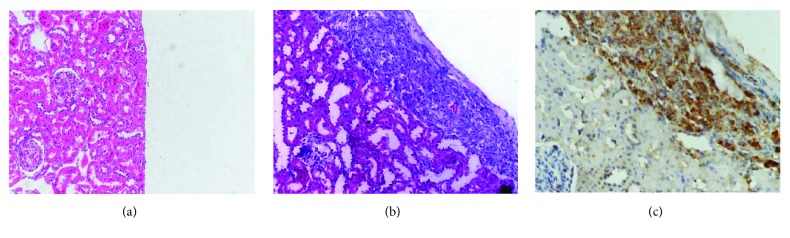
Evaluation of islet transplantation. (a) Normal renal HE staining. (b) IT group renal HE staining (200x). (c) Anti-insulin immunohistochemistry (400x).

Figure 1 shows that the blood glucose in the IT group returned to 7.5 mmol/L, while its weight increased significantly 2 weeks following islet transplantation (IT). Compared with the DN group, the urine protein-to-creatinine and microalbumin-to-creatinine ratios of the normal group significantly decreased (*p* < 0.001). In Figure 2, the results of renal HE and islet transplantation staining show pancreatic islet cells under the renal capsule.

### 3.3. Evaluation of Renal Function

Figures 1(e) and 1(f) show that the SCR (49 ± 2.72 *μ*mol/L) and the BUN (33.2 ± 10.6 mmol/L) in the DN group increased significantly compared with those of the normal group (23.94 ± 1.4 μmol/L and 5.8 ± 0.74 mmol/L, resp.) (*p* < 0.001 and *p* < 0.01, resp.). Compared with the DN group, the SCR and BUN of the IT group (23.1 ± 5.3 *μ*mol/L and 7.38 ± 2.02 mmol/L, resp.) (*p* < 0.001 and *p* < 0.01, resp.) significantly decreased. These dates showed that the islet transplantation improved the kidney in early diabetic nephropathy rats.

### 3.4. Expression of PTX3 in Renal Tissue

**Figure 3:**
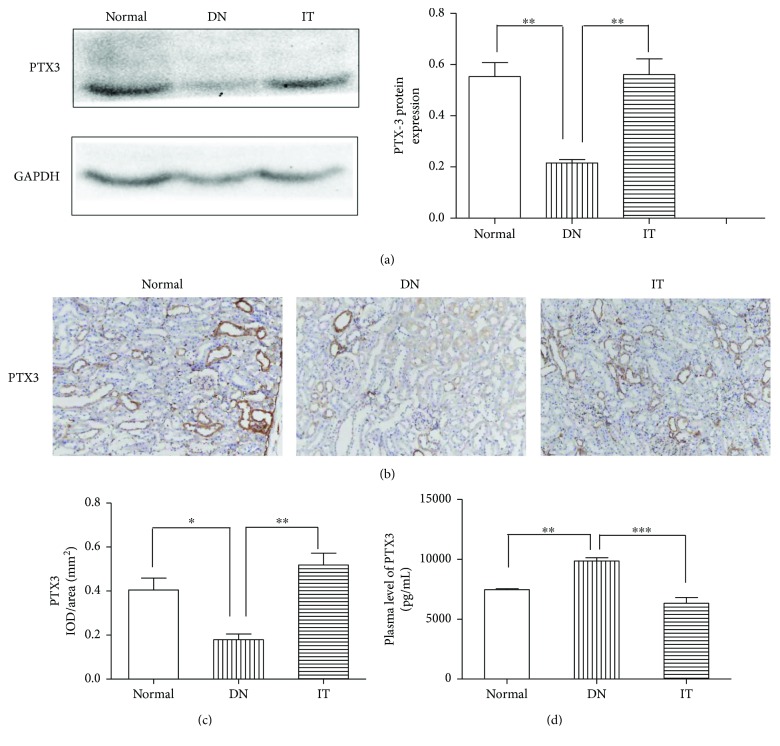
The expression of PTX3 in each group. (a) The expression of PTX3 protein as detected by Western blot. (b, c) The expression of PTX3 protein as detected by immunohistochemistry. (d) The plasma PTX3 concentration. ^∗^*p* < 0.05, ^∗∗^*p* < 0.01, and ^∗∗∗^*p* < 0.001.

The expression of PTX3 in the kidney was evaluated with Western blot and immunohistochemistry. In Figure 3(a), Western blot showed that PTX3 expression in the DN group was significantly lower than in the normal group (*p* < 0.01), while PTX3 expression in the IT group was significantly higher than in the DN group (*p* < 0.01). Further, PTX3 expression in the IT group was closest to that of the normal group. In Figures 3(b) and 3(c), immunohistochemistry showed that the expression of PTX3 in the DN group was lower than that in the normal group (*p* < 0.05), while PTX3 expression in the IT group was significantly higher than that in the DN group (*p* < 0.01). PTX3 expression in the kidneys of the normal group was mainly located in renal tubular epithelial cells.

### 3.5. Expression of PTX3 in Plasma

The expression of plasma PTX3 was evaluated by enzyme-linked immunosorbent assay (ELISA). In Figure 3(d), mean plasma ptx3 in the DN group (9851.4 ± 808.6 pg/mL) was significantly higher compared to those in the normal group (7457.7 ± 158.5 pg/mL) and IT group (6314.6 ± 1086.4 pg/mL) (*p* < 0.01 and *p* < 0.001, resp.).

### 3.6. Assessment of Renal Injury

**Figure 4:**
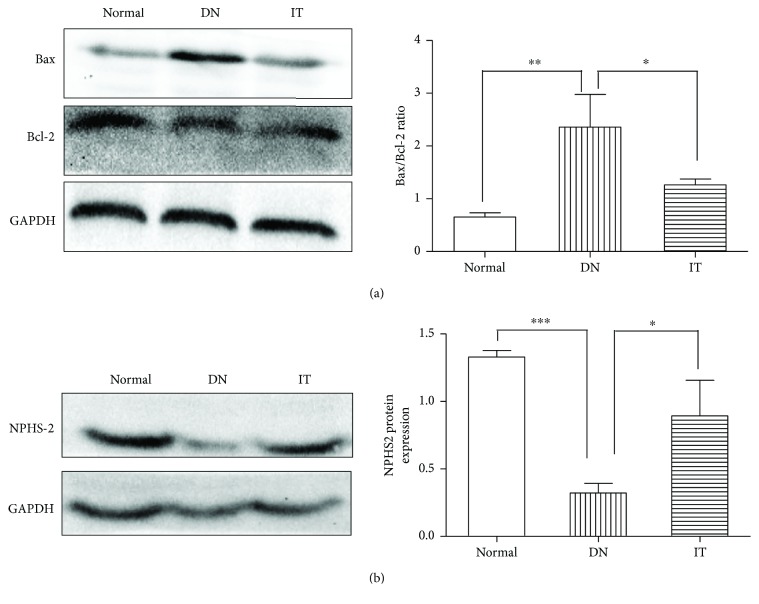
Western blot analysis of Bax, Bcl-2, and NPHS-2 in the renal tissues in each group. (a) Bax and Bcl-2. (b) NPHS-2. ^∗^*p* < 0.05, ^∗∗^*p* < 0.01, and ^∗∗∗^*p* < 0.001.

**Figure 5:**
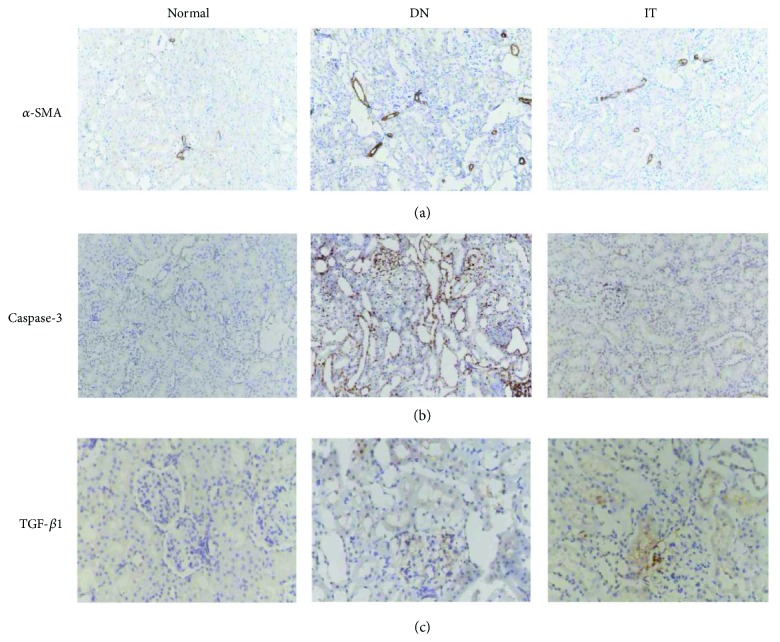
Immunohistochemistry was used to detect the expression of renal fibrogenic and interstitial fibrosis factors and apoptotic proteins. (a) *α*-SMA. (b) Caspase-3. (c) TGF-*β*1.

**Figure 6:**
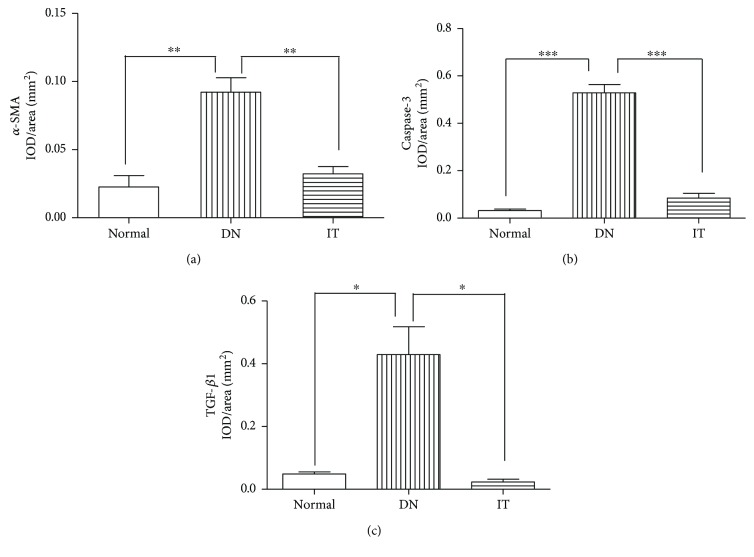
Measurement of the IOD/area of immunohistochemical staining of renal fibrogenic and interstitial fibrosis factors and apoptotic proteins. (a) Measurement of the IOD/area of *α*-SMA, ^∗∗^*p* < 0.05. (b) Measurement of the IOD/area of caspase-3, ^∗^*p* = 0.018 and ^∗∗∗^*p* < 0.001. (c) Measurement of the IOD/area of TGF-*β*1, ^∗^*p* < 0.05. IOD/area: integrated optical density/area. The mean of IOD/area of positive staining was used to accurately measure the degree of positive expression in immunohistochemistry samples.

Immunohistochemistry and Western blots were used to evaluate the degree of renal injury in each group. In Figure 4(a), the Bax/Bcl-2 ratio of the DN group was higher than that of the normal and IT groups (*p* < 0.01 and *p* < 0.05, resp.). In Figure 4(b), the expression of NPHS-2 in the DN group was lower than that of the normal and IT groups (*p* < 0.001 and *p* < 0.05, resp.). In Figures 5 and 6, the expression of *α*-SMA, cleaved caspase-3, and TGF-*β*1 in the DN group was significantly higher than that in the normal and IT groups (*p* < 0.01, *p* < 0.001, and *p* < 0.05, resp.).

## 4. Discussion

At present, there are approximately 300 million diabetic patients in the world, a figure that will likely reach 500 million by 2030 [21]. Approximately 20–40% of diabetic patients develop diabetic nephropathy [22]. As one of the most common microvascular complications of diabetes, the prevalence of DN has had a rapid growth trend. The prevention and diagnosis of renal injury in early diabetic nephropathy can help improve the life expectancy, quality of life, and economic burden of diabetic patients.

PTX3 is the first cloned long pentraxin that can be detected in renal tissue [13]. The relationship between PTX3 and various acute and chronic kidney diseases is a hot topic. The levels of plasma PTX3 in early acute ischemia-reperfusion renal injuries and chronic kidney disease are response to be increased [23, 24], and the expression of PTX3 in the renal tubular epithelial cells during ischemia-reperfusion kidney injuries and the glomerular endothelial cells in patients with IgA nephropathy [13] and type I membranous proliferative glomerulonephritis were increased. Hence, PTX3 was previously thought to have a potential pathogenic effect as a proinflammatory cytokine [25, 26]. However, in recent years, an increasing number of studies have shown that PTX3 does not cause kidney damage [27] and that an elevated plasma PTX3 concentration is likely a marker of immune inflammatory response and is a physiologic protective defense mechanism, with an anti-inflammatory and protective effect on the kidneys. Previous studies have demonstrated that PTX3 can limit inflammation, promote the repair/regeneration of damaged tissue [28], and induce and maintain immune tolerance. PTX3 binds P-selectin, which allows white blood cells to roll into the apoptotic membrane of activated microvascular endothelial cells, thereby limiting subsequent leukocyte adhesion and cross-endothelial migration. This would limit the recruitment of neutrophils and macrophages in the ischemic kidney [14], stimulating tubular repair [27]. Lech et al. [14] noted that PTX3-deficient mice had worse ischemic acute kidney injuries, which were improved following recombinant PTX3 injection. Another study [29] showed that PTX3 treatment inhibited interstitial fibrosis induced by an acute kidney injury, reduced serum creatinine levels, and decreased the expression of collagen and smooth muscle actin in mice.

In our study, the expression of PTX3 in the kidney was mainly located in renal tubular epithelial cells and was significantly decreased in our DN model. To further explore the relationship between PTX3 and DN, islet transplantation was performed in DN rats to treat diabetic nephropathy. Here, we provide the first evidence that the expression of PTX3 in the IT group was significantly greater than in the DN group and was similar to normal animals. But the expression of plasma PTX3 was though contrary to the above results. In the recent studies, the concentration of plasma PTX3 has been reported as a close and independent determinant of proteinuria, impaired kidney function, and endothelial dysfunction [24, 30]. The increased level of plasma PTX3 in the DN group showed that the kidney function is damaged and the endothelial cell is dysfunction. The results of SCR and BUN proved this conclusion in our study. However, plasma PTX3 of the rats in the IT group is lower as compared to that of the DN group, demonstrating that the renal function and the endothelial cells are restored. All of the aforementioned evidence suggests that PTX3 is involved in the development of DN. However, its mechanism of action still remains unclear.

Previous studies have found that renal tubule cell apoptosis and renal interstitial fibrosis play an important role in the development of DN beyond glomerular filtration barrier damage and glomerular sclerosis [31]. Renal tubule lesions occur earlier than glomerular lesions and are closely related to the extent and severity of renal dysfunction [31]. In hyperglycemic conditions, cell culture and animal experiments showed a significant amount of renal tubular epithelial cell apoptosis [32]. The proapoptotic protein bax and the antiapoptotic protein bcl-2, which belong to the bcl-2 family, are very important regulatory factors in the apoptotic pathway. Caspase-3, which cleaves DNA-dependent protein kinases to destroy cell structure proteins, is the most important proapoptotic protease during apoptosis [33]. The present study demonstrated that the expression of Bax/Bcl-2 in the DN group was higher than in the normal group. The immunohistochemistry staining of cleaved caspase-3 showed that there was a large amount of renal tubular epithelial cell apoptosis in the DN model. Further, the expression of Bax/Bcl-2 and cleaved caspase-3 was reversed after islet transplantation. These data suggest that the apoptosis of renal tubular epithelial cells is linked to DN. According to the expression of PTX3 in the kidney, we therefore hypothesize that renal tubular epithelial cell apoptosis and progressive loss result in the gradual decline of PTX3 synthesis and secretion. Furthermore, PTX3 is an important contributor to the development of cell apoptosis. Soluble and membranous forms of PTX3 can independently prevent and promote the autoimmune process, suggesting that PTX3 has a dual effect and that its function may be dependent on the environment, treatment structure, cytokine spectrum, and gene mutations [34]. Under infectious and inflammatory conditions, PTX3 regulates the autoimmune response, induces apoptosis, and promotes the leakage of cellular contents. By controlling the interaction between mature dendritic cells and apoptotic cells, PTX3 participates in the removal of apoptotic cells [12, 35]. Under inflammatory and other conditions, PTX3 induces the activation of the classical complement pathway, specifically binding apoptotic cells and cell debris on the surface of complement component C3b/iC3b, promoting complement-mediated apoptosis and cell clearance [28]. The acceleration of cellular apoptosis in DN led to a further decrease in PTX3 expression.

We detected an elevated TGF-*β*1 and *α*-SMA expression and decreased NPHS-2 (podocin) expression in the DN group as compared with the normal and IT groups. TGF-*β*1 and *α*-SMA are the major factors in the progression of renal fibrosis in DN. NPHS-2 (podocin), a protein encoded by the NPHS-2 gene, interacts with nephrin and CD2AP, an important component of the membrane, in the same way as the glomerular filtration barrier [36]. The reduction expression of NPHS-2 may thus lead to proteinuria [37]. On the basis of these findings, we found that the level of PTX3 in renal tissue was closely related to renal injury in DN. But the mechanism how the PTX3 influence the renal injury in DN is still unclear. Maybe we will investigate the reasons in the future.

In conclusion, the present study found that the level of PTX3 in renal tissue is closely related to renal injury in DN. The expression of PTX3 in the kidney was significantly decreased in the DN group, while it was increased in the IT group because of the reversal of DN. This may be used to quantify the extent of renal injury in DN, provide a potential indicator in the early assessment of renal tubule injury in early DN patients, and assess DN clinical progression. This also could provide better advice for clinical treatment in diabetes patients to prevent the development and progress of DN.
